# DN2 Thymocytes Activate a Specific Robust DNA Damage Response to Ionizing Radiation-Induced DNA Double-Strand Breaks

**DOI:** 10.3389/fimmu.2018.01312

**Published:** 2018-06-11

**Authors:** Irene Calvo-Asensio, Tara Sugrue, Nabil Bosco, Antonius Rolink, Rhodri Ceredig

**Affiliations:** ^1^National University of Ireland, Galway, Ireland; ^2^Department of Biomedicine, University of Basel, Basel, Switzerland

**Keywords:** DN2 pro-T cells, DNA damage response, ionizing radiation, hypoxia, thymic auto-reconstitution, bone marrow transplantation

## Abstract

For successful bone marrow transplantation (BMT), a preconditioning regime involving chemo and radiotherapy is used that results in DNA damage to both hematopoietic and stromal elements. Following radiation exposure, it is well recognized that a single wave of host-derived thymocytes reconstitutes the irradiated thymus, with donor-derived thymocytes appearing about 7 days post BMT. Our previous studies have demonstrated that, in the presence of donor hematopoietic cells lacking T lineage potential, these host-derived thymocytes are able to generate a polyclonal cohort of functionally mature peripheral T cells numerically comprising ~25% of the peripheral T cell pool of euthymic mice. Importantly, we demonstrated that radioresistant CD44^+^ CD25^+^ CD117+ DN2 progenitors were responsible for this thymic auto-reconstitution. Until recently, the mechanisms underlying the radioresistance of DN2 progenitors were unknown. Herein, we have used the *in vitro* “*Plastic Thymus*” culture system to perform a detailed investigation of the mechanisms responsible for the high radioresistance of DN2 cells compared with radiosensitive hematopoietic stem cells. Our results indicate that several aspects of DN2 biology, such as (i) rapid DNA damage response (DDR) activation in response to ionizing radiation-induced DNA damage, (ii) efficient repair of DNA double-strand breaks, and (iii) induction of a protective G_1_/S checkpoint contribute to promoting DN2 cell survival post-irradiation. We have previously shown that hypoxia increases the radioresistance of bone marrow stromal cells *in vitro*, at least in part by enhancing their DNA double-strand break (DNA DSB) repair capacity. Since the thymus is also a hypoxic environment, we investigated the potential effects of hypoxia on the DDR of DN2 thymocytes. Finally, we demonstrate for the first time that *de novo* DN2 thymocytes are able to rapidly repair DNA DSBs following thymic irradiation *in vivo*.

## Introduction

In adults, the bone marrow is the main organ in which hematopoiesis takes place. There, self-renewing, multipotent hematopoietic stem cells (HSCs) reside in a specialized niche and are responsible for continually giving rise to all types of hematopoietic cells (1–4). However, unlike other hematopoietic cells, T lymphocytes are not produced in the bone marrow but rather in the thymus (5, 6), an organ that provides the optimal microenvironment for supporting all stages of T cell development and selection (7, 8). Under normal physiological conditions, the thymus does not contain self-renewing HSCs. Instead, it is continuously seeded by bone marrow-derived multipotent progenitors that migrate there through the blood (9, 10). Once within the thymus, these progenitors receive signals from the thymic stroma that induce them to proliferate and to undergo progressive differentiation into mature, functional, and self-tolerant T cells (11, 12).

Within the thymus, developing T cells, i.e., immature thymocytes, undergo a series of developmental stages that can be distinguished according to the surface expression pattern of the CD4 and CD8 co-receptors. In a normal thymus, ~5% of thymocytes express neither CD4 nor CD8 (double-negative, DN cells); ~80% express both CD4 and CD8 (double-positive, DP cells); ~10% are CD4 single-positive (CD4SP), and ~5% CD8 single-positive (CD8SP) (13). The most immature intra-thymic T cell progenitors are contained within the DN population (14) and according to their surface expression of CD25, CD44, and CD117, DN cells can be further subdivided into four major cellular subsets known as DN1 to DN4. DN1 cells, the most undifferentiated DN subset, can be identified as CD25^−^CD44^+^CD117^+^; DN2 cells are CD25^+^CD44^+^CD117^+^; DN3 cells are CD25^+^CD44^low^CD117^low^, and finally DN4 cells, the most differentiated DN subpopulation, are negative for all three markers (CD25^−^CD44^−^CD117^−^) (10, 14).

Upon irradiation, thymic cellularity is dramatically reduced due to the high radiosensitivity of thymocytes (15). However, shortly after exposure to a lethal dose of ionizing radiation (IR), unlike all other hematopoietic and lymphoid organs, there is a single wave of thymic auto-reconstitution that results from the proliferation and differentiation of host-derived intra-thymic radioresistant T cell precursors (16–19). These cells would also appear to be resistant to the administration of hydrocortisone acetate (20) and were identified by Bosco et al. as DN2 thymocytes (21). These DN2 thymocytes were able to give rise to a cohort of functional mature T lymphocytes capable of re-constituting ~25% of the normal peripheral T cell compartment, and displaying a polyclonal T cell receptor (TCR) repertoire (21). However, the reasons why specifically these DN2 thymocytes are able to survive following thymic irradiation and subsequently resume their normal intra-thymic development post-IR is currently unknown. Therefore, further investigation is required to define the molecular mechanisms underlying the radioresistance of DN2 thymocytes.

Historically, different systems have been developed to study T cell development, including (i) fetal thymus organ culture (FTOC), (ii) re-aggregated FTOC, (iii) bone marrow chimeras, and (iv) transgenesis (22, 23). More recently, *in vitro*-based expansion and differentiation of immature thymocytes using stromal cell lines ectopically expressing Notch ligands have been used to dissect the signaling events required for T cell development (24–27). However, in these *in vitro* systems, the exact combination and intensity of signals delivered by stromal cells are difficult to control. In addition, the presence of stromal cells in these cultures makes detailed genetic and molecular analysis of uniquely T cell-specific events occurring within cultured progenitors difficult to dissect. The recent development of a stromal cell-free pro-T cell culture system in the laboratory of Prof. Antonius Rolink has proven to be a very useful tool for studying the minimal requirements necessary for T-cell commitment and differentiation (28). This stromal cell-free culture system commonly known as “*The Plastic Thymus*” is based on the immobilization of a DL4-human IgG_1_-Fc (DL4-Fc) fusion protein to the surface of plastic tissue culture plates pre-coated with a monoclonal anti-human IgG_1_-Fc antibody (28). In addition, the culture medium is supplemented with IL-7 and SCF, allowing the long-term *in vitro* maintenance and expansion of purified DN2 thymocytes (29). Importantly, the pro-T cells generated and expanded *in vitro* using this methodology (i) retain their normal functionality, (ii) can be genetically manipulated, and (iii) are able to reconstitute T cell compartments of irradiated recipient mice (29). Therefore, “*The Plastic Thymus*” represents a novel technology with which to study purified DN2 thymocytes at the molecular level, something that is otherwise technically difficult to do in the normal mouse thymus *in vivo* due to the limited numbers of pro-T cells, particularly DN1 and DN2 cells.

Cellular responses to IR exposure mainly occur due to its detrimental impact on the genome integrity of exposed cells. IR-induced DNA damage can occur due to energy deposited directly onto DNA, or indirectly due to the generation of free radicals within cells, which collectively lead to the modification and/or breakage of DNA strands. The most genotoxic IR-induced DNA lesions are DNA double-strand breaks (DNA DSBs). The maintenance of genomic integrity is essential for cellular survival and for preventing carcinogenesis. Consequently, cells have developed an integrated series of signaling networks, known collectively as the DNA damage response (DDR), to mount biological responses to genotoxic insult. At the molecular level, the DDR consists of (i) sensor proteins that recognize sites of damaged DNA, (ii) transducer proteins that amplify DNA damage signals, and (iii) effector proteins, required for the desired biological response(s) including DNA repair, transient delays in cell cycle progression (termed checkpoints), transcriptional and epigenetic programs, apoptosis, and senescence (30, 31).

Similar to DN2 thymocytes, mesenchymal stromal cells (MSCs) that support hematopoiesis in the bone marrow, and thymic epithelial cells (TECs), which support thymopoiesis in the thymus, are also relatively radioresistant (32–34). In previous studies, we have demonstrated that the activation of the DDR plays important roles in enabling *in vitro* irradiated MSCs and TECs to quickly respond to IR-induced DNA damage and to engage molecular pathways that promote rapid DNA DSB repair, DNA damage checkpoint activation, and cell survival (34, 35). Therefore, using the methods we have previously applied to investigate the DDR of irradiated MSCs, we aimed herein to investigate the role of the DDR in mediating the radioresistance of DN2 thymocytes.

In this study, we have used the “*Plastic Thymus*” culture system to dissect DN2 radiobiology at the molecular level. For comparative purposes, and as a model of a radiosensitive hematopoietic cell type, we have also used a NUP98-HOXB4 expressing HSC line (refer to Section “Materials and Methods” for further information). We demonstrate for the first time that in response to IR-induced DNA DSBs, DN2 thymocytes execute a robust DDR leading to the resolution of these lesions and to cell survival. Furthermore, we also demonstrate that this robust DDR is also executed by DN2 thymocytes *in vivo* following thymic irradiation. Taken together, our results from both *in vitro* and *in vivo*-derived cells indicate that the DDR of DN2 thymocytes harbors characteristic features favoring their ability to rapidly respond to IR-induced DNA damage and repair DNA lesions, likely playing a key role in their relative radioresistance.

## Materials and Methods

### Cell Culture and Treatments

DN2 thymocytes (CD4^−^ CD8^−^ CD44^+^ CD25^+^ c-kit^+^ CD127^+^) were isolated from the thymi of 4- to 6-week-old C57BL/6 mice as previously described (10) and sorted using a BD FACS Aria^®^ cell sorter. As previously discussed, the “*Plastic Thymus*” culture system allows long-term expansion of DN2 pro-T cells *in vitro* in the absence of stromal cells (29). Details of this culture system are described in Section Supplementary Methods in Supplementary Material and shown graphically in Figure S1 in Supplementary Material. DN2 thymocytes were shown to maintain their characteristic cell surface phenotype (CD44^+^ CD25^+^ CD117^+^) when cultured long-term in 21% O_2_ (Figure S2 in Supplementary Material).

To study HSCs, a NUP98-HOXB4 HSC (NH-HSC) line was generated from C57BL/6 mice following the protocol established by Sauvageau et al. (36) and subsequently optimized by Ruedl et al. (37). The NH-HSC line obtained following this protocol was confirmed to display the surface phenotype: CD45^+^ Lin^−^ c-kit^+^ Sca-1^+^ CD11c^−^ CD19^−^ B220^−^ CD4^−^ CD8^−^ and to be capable of successfully re-constituting all hematopoietic lineages in sub-lethally irradiated mice (38). For the purposes of this study, NH-HSCs were maintained in SF-IMDM (Gibco) supplemented with 5% forward light scatter (FCS) (Gibco), 3% v/v IL-6-containing supernatant, 0.1 µg/ml SCF and 0.2% v/v Ciproxin^®^ (Bayer Pharmaceutical) at 37°C [as described in Ref. (38)], in either normoxia (21% O_2_) or hypoxia (5% O_2_).

γ-Irradiation at the indicated doses was performed using a Gammacell 40 irradiator containing a ^137^Cs source at a dose rate of ~80 cGy/min.

### Mice

C57BL/6 mice were bred under pathogen-free conditions at the Centre for Biomedicine at the University of Basel. All animal experiments were carried out within institutional guidelines (authorization numbers 1886 and 1888 from Kantonales Veterinäramt, Basel).

### Isolation and Sorting of Mouse CD4/CD8 DN 1–3 Subpopulations

Double-negative cells were isolated from 5 thymi per time point after irradiation (9 Gy) and sorted according to their cell surface phenotypes as previously described (10) and outlined in Section Supplementary Methods and Figure S3 in Supplementary Material. DN1 were sorted as CD117^high^, CD25^−^ and CD44^high^, DN2 as CD117^high^, CD25^+^ and CD44^high^ and DN3 as CD117^low^, CD25^+^ and CD44^low^ (Figure S3 in Supplementary Material) (10). Sorted cells were pelleted, re-suspended in 100 µl IMDM, and centrifuged onto poly-l-lysine-coated microscope slides using a Cytospin^®^ centrifuge (Shandon) for immunofluorescence staining as described below.

### Clonogenic Survival Assay

DN2 and NH-HSC were irradiated at 0.5–4 Gy, seeded into 6-well plates at a concentration of 50,000 cells/well, harvested 3 or 5 days post-irradiation (for NH-HSCs and DN2 cells, respectively) and viable cell numbers were counted in duplicate using a hemocytometer and Trypan blue for exclusion of dead cells. The time-points for cell counting were selected based on the fact that DN2 thymocytes proliferated more slowly than NH-HSCs (Figure S4 in Supplementary Material). The percentage survival of each cell type was then determined by normalizing the number of cells quantified from irradiated versus control (un-irradiated) cultures.

### Flow Cytometry Methods

Cells were harvested and counted prior to staining following the different protocols described below. Cells were then analyzed using a BD FACS Canto^®^ or BD FACS Calibur flow cytometer (BD Biosciences) and FlowJo^®^ data analysis software (Tree Star Inc., OR, USA). For surface labeling, cells were pelleted and re-suspended in FACS buffer (2% FCS, 0.05% Sodium Azide, PBS) at 5 × 10^6^ cells/ml. Then, 5 × 10^5^ cells/sample were stained for 20 min with the appropriate primary antibodies or isotype controls. To reveal cells stained with biotin-labeled antibodies, the cells were subsequently washed in FCS-free FACS buffer, stained for 10 min with fluorescently labeled streptavidin, washed, and then analyzed.

For cell cycle analysis, cells were labeled for 1 h with 25 µM 5′-bromo-deoxyuridine (BrdU) (Sigma-Aldrich), washed with PBS, and re-suspended in growth medium. Cells were harvested at the indicated time points post-irradiation (4 Gy), fixed in ice-cold 70% ethanol, and stained with anti-BrdU and FITC-conjugated anti-mouse IgG antibodies and propidium iodide (PI)/RNase staining buffer (BD Biosciences) as previously described (35). The progression of cells through the cell cycle was analyzed by measuring the percentage BrdU-positive cells in each G_1_ phase until 24 h post IR using a BD FACS Canto^®^ flow cytometer (BD Biosciences) and FlowJo^®^ software (Tree Star Inc., OR, USA).

Information regarding all antibodies used for flow cytometry can be found in Section Supplementary Methods in Supplementary Material.

### Real-Time PCR

Total RNA was isolated from cells by TRIzol^®^ Reagent (Life Technologies)—and cDNA was generated using Applied Biosystems’ High-Capacity cDNA Reverse Transcription Kit according to the manufacturer’s instructions. 10–20 ng of cDNA was used as template in semi-quantitative real-time PCR reactions with specific primers on a Step One Plus Real-Time PCR System (Applied Biosystems). Reactions were prepared with TaqMan^®^ gene expression master mix (Thermo Fisher Scientific) using predesigned TaqMan^®^ gene expression assays for amplification of mouse Lig4 (DNA Ligase 4), Prkdc (DNA-PKcs), Rad51, and β-actin (Thermo Fisher Scientific). Gene expression changes were determined using the ΔΔCt method, using β-actin as a housekeeping gene for normalization according to the primer efficiencies previously calculated.

### Western Blotting

Whole cell extracts were prepared from control or irradiated cells at the indicated time points post-irradiation by direct addition of 5 µl of 4× Laemmli buffer per 100,000 cells that we previously harvested from culture by centrifugation, washed with ice-cold PBS, and counted. Cells were disaggregated into the Laemmli buffer, heated at 95°C for 5 min, and sonicated (20% amplitude for 3 s) prior to separation using SDS-PAGE gels and transferred to nitrocellulose membranes. Chemiluminescence was detected using SuperSignal West Pico Chemiluminescent Substrate (Thermo Fisher Scientific) and medical x-ray film (Konica Minolta Medical and Graphic Imaging Inc.). Information regarding all antibodies used for western blotting can be found in Section Supplementary Methods in Supplementary Material.

### Immunofluorescence Staining and Microscopy

Double-negative cells were spun onto poly-l-lysine-coated microscope slides using a Cytospin^®^ centrifuge (Shandon), fixed in 4% paraformaldehyde (Sigma-Aldrich), and subsequently permeabilized in 0.1% Triton^®^-X 100 solution. Nuclei were then stained for γH2AX IR-induced foci (IRIF) as previously described (35). Image Z-stacks were captured using 60× magnification on a Leica SP5 integrated microscope system (Leica Microsystems). Images were deconvoluted using the Huygens Software by Scientific Volume Imaging. Image Z-stacks were projected using the maximal intensity method using Fiji (39). Customized macros for *Fiji* were used to adjust the projected images and the number of γH2AX IRIF per nucleus were counted blind. Information regarding all antibodies used for western blotting can be found in Section Supplementary Methods in Supplementary Material.

## Results

### DN2 Thymocytes Are Relatively Radioresistant *In Vitro*

**Figure 1:**
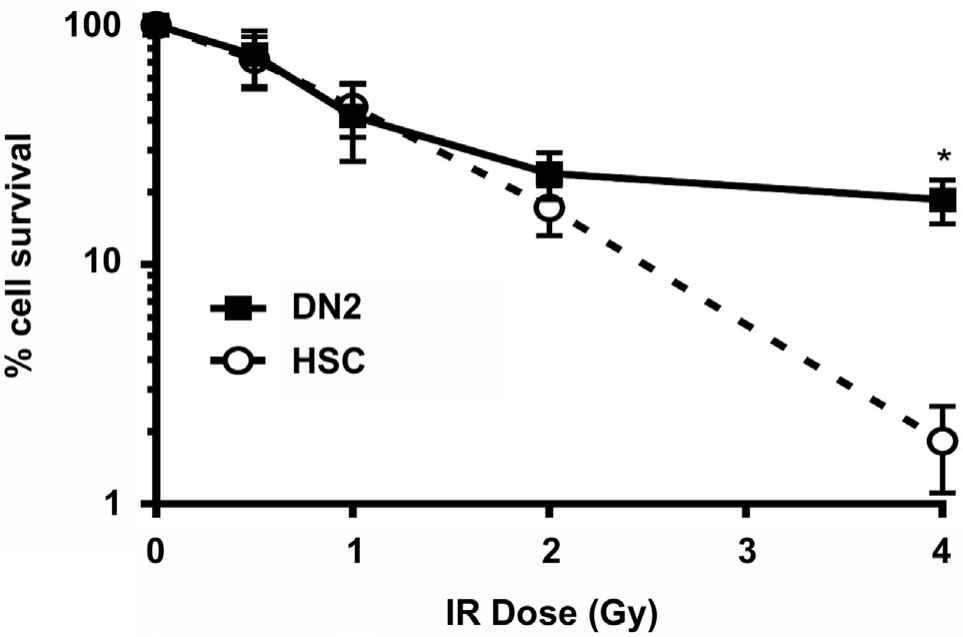
Double-negative (DN)2 thymocytes survive γ-irradiation *in vitro*. Clonogenic survival assays of mouse DN2 thymocytes and NH-hematopoietic stem cells (HSCs) γ-irradiated at 0.5–4 Gy and cultured for 3 or 5 days (depending on the cell type). Error bars represent mean ± SD, *n* = 3. *p < 0.05, two-way ANOVA analysis with Sidak’s multiple comparisons test.

We first investigated the effects of irradiation on the survival of DN2 thymocytes *in vitro*, using NH-HSCs as a radiosensitive control. To do so, DN2 and NH-HSC were irradiated at 0–4 Gy and cultured for 3 (NH-HSC) or 5 days (DN2) before counting surviving cells. As shown in Figure 1, the radioresistance of DN2 and NH-HSC was comparable at IR doses of 0.5–2 Gy. However, at 4 Gy, the radioresistance of DN2 cells was found to be 10-fold greater than that of NH-HSC (~18.7% DN2 survival versus ~1.8% NH-HSC survival) (Figure 1).

### DN2 Thymocytes Activate DNA Damage Checkpoints *In Vitro*

**Figure 2:**
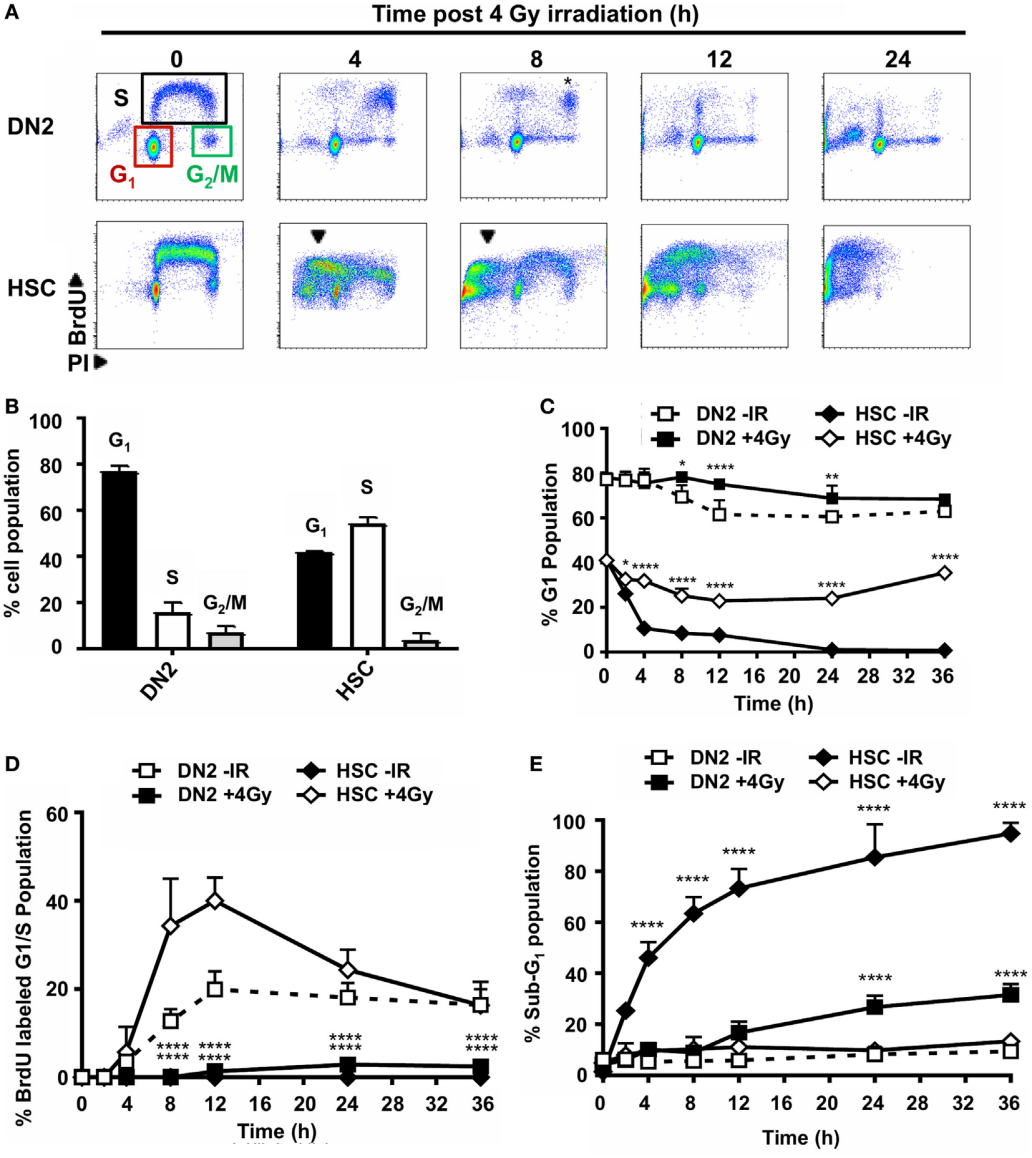
Double-negative (DN)2 thymocytes activate DNA damage checkpoints. **(A)** Representative cytograms of DN2 and NH-hematopoietic stem cells (HSCs) at 0–24 h post 4 Gy irradiated and stained for bromo-deoxyuridine (BrdU) incorporation and DNA content using propidium iodide (PI). Colored boxes are used to indicate G_1_, S, and G_2_/M phase cells under control conditions; black arrowheads indicate sub-G_1_ cells and * indicates cohort of BrdU-labeled DN2 cells accumulated in late S/G_2_. **(B)** Quantification of the percentage DN2 and NH-HSCs in each phase of the cell cycle under normal conditions. **(C)** Quantification of the percentage G_1_ DN2 cells and NH-HSCs at 0–36 h post-irradiation or under normal conditions. **(D)** Quantification of the percentage BrdU-labeled G_1_/early S phase DN2 cells and NH-HSCs at 0–36 h post-irradiation or under normal conditions. **(E)** Quantification of the percentage sub-G_1_ DN2 cells and NH-HSCs at 0–36 h post-irradiation or under normal conditions All cytograms and graphs are representative of three independent experiments. Error bars represent mean ± SD, *n* = 3, *p < 0.05, **p < 0.01, ****p < 0.0001. Two-way ANOVA analysis with Tukey’s multiple comparisons test was performed on the following conditions (i) DN2-ionizing radiation (IR) versus DN2 + 4 Gy and (ii) HSC-IR versus HSC + 4 Gy.

As previously mentioned, the DDR plays a key role in determining whether a cell survives or dies following exposure to genotoxic agents, such as IR. The activation of DNA damage checkpoints during the cell cycle acts to prolong the time in which a given cell can execute mechanisms to try and resolve genomic damage and promote its survival. The difference in the long-term survival between DN2 and NH-HSC suggested that these DNA damage checkpoints may be differentially executed between these two cell types. To investigate this, the cell cycle progression of BrdU pulse-labeled DN2 and NH-HSCs was analyzed at various time points post 4 Gy irradiation by flow cytometry (Figure 2A). By co-staining these cells with an anti-BrdU antibody and PI, the G_1_, S, and G_2_/M phases of the cell cycle (Figure 2A, top left panel) can be clearly distinguished. Furthermore, the progression of BrdU-labeled (S phase) cells through the cell cycle and their return to the G_1_ phase can be monitored (Figure 2A). Importantly, under control conditions (no irradiation, 0 Gy), BrdU labeling itself did not cause significant toxicity to either DN2 or NH-HSCs (Figure S4 in Supplementary Material). Under normal conditions (0 h), ~16% DN2 population were in S phase (BrdU-positive) compared with ~56% NH-HSC (Figure 2B). Furthermore, ~78% DN2 population was found to be in G_1_ phase, compared with ~41% NH-HSC population (Figure 2B). These results correlate with the increased rate of cell cycle progression observed in NH-HSCs, compared with DN2 cells (Figure S4 in Supplementary Material).

Dramatic differences in the ability of DN2 and NH-HSC to activate cell cycle arrest were observed post-irradiation. Strikingly, DN2 thymocytes that were in G_1_ phase at the time of irradiation were largely maintained over time (~78% in G_1_ at 0 h versus ~68.4% in G_1_ at 36 h post IR) (Figure 2C). This strongly contrasted with NH-HSCs whose G_1_ population reduced rapidly post-irradiation from ~41% at 0 h to ~0.8% at 36 h post IR (Figure 2C). This reduction in % G_1_ cells was likely due to the activation of cell death as evidenced by the appearance of a large sub-G_1_ population in NH-HSC profiles post-irradiation (Figure 2E and indicated in Figure 2A by black arrows in bottom panels). BrdU-labeled DN2 cells accumulated as a cohort in late S/G_2_ phases until 8 h post IR, indicative of the activation of intra-S-phase and G_2_ checkpoints (32) (Figure 2A, * in top 8 h panel). However, BrdU labeled DN2 thymocytes did not re-enter cell cycle following G_2_ checkpoint activation and likely entered cell death, similar to NH-HSCs (Figure 2B, top 24 h panel). Similar to the G_1_ NH-HSC population, BrdU labeled NH-HSCs also underwent cell death post IR (Figures 2A,E). Taken together, these results indicate that in the immediate response to irradiation, DN2 thymocytes activate DNA damage checkpoints more robustly than NH-HSCs which instead seem to directly revert to activating cell death. Interestingly, these results also demonstrate that DN2 thymocytes that survive long-term post IR originate primarily from cells in G_1_ phase of the cell cycle, indicating that DN2 thymocytes induce a protective G_1_/S checkpoint in response to IR-induced DNA damage.

### DN2 Thymocytes Activate a Robust DDR Post-Irradiation

**Figure 3:**
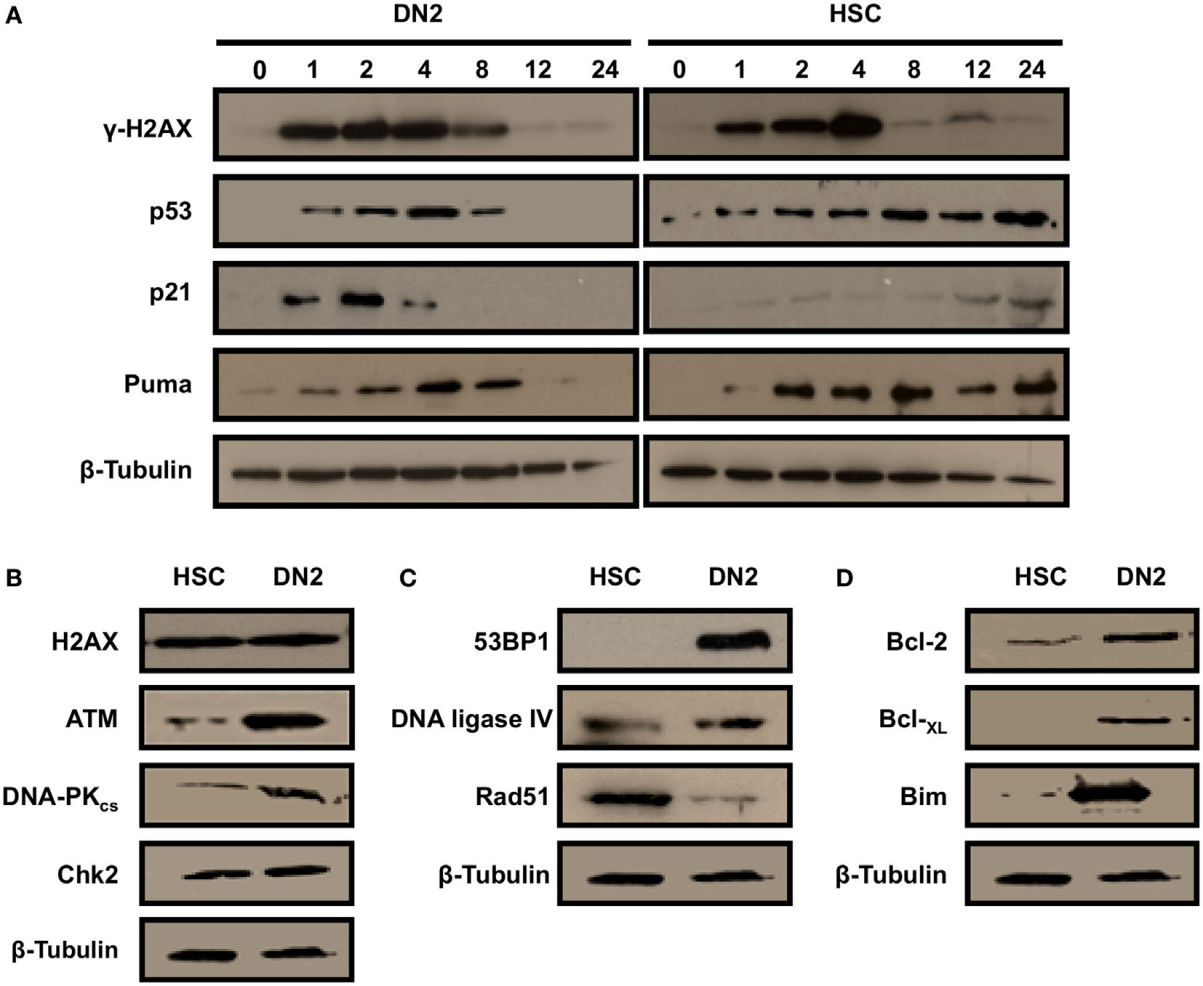
DN2 thymocytes activate a robust DNA damage response following γ-irradiation. Western blot analysis of **(A)** H2AX (Ser139) phosphorylation (γH2AX—marker of DNA double-strand breaks), p53 stabilization; and p21 and Puma expression in DN2 cells and NH-hematopoietic stem cells (HSCs) at 0–24 h post 4 Gy irradiation. Western blot analysis of total endogenous levels of **(B)** of H2AX, ATM, DNA-PKcs and Chk2; **(C)** of 53BP1, DNA ligase IV and Rad51; and **(D)** of Bcl-2, Bcl-XL, and Bim in un-irradiated (control) DN2 cells and NH-HSCs. β-Actin and β-tubulin were used as internal controls. All images are representative of one of three independent experiments.

The contrasting cellular responses of DN2 thymocytes versus NH-HSCs to IR-induced DNA Damage (strong induction of G_1_ arrest in irradiated DN2 thymocytes versus apoptosis in NH-HSCs) suggested that the molecular DDR pathways may be differentially executed in these two cell types. To determine whether DN2 and HSC differentially activate the DDR in response to IR-induced DNA DSBs, H2AX Ser139 phosphorylation (γH2AX, DNA DSB marker); p53 stabilization; and p21 and Puma expression were analyzed over a 24-h time-course (Figure 3A). Maximal H2AX phosphorylation was detected in DN2 at 1 h post IR, whereas it was delayed in HSC and accumulated until 4 h post IR (Figure 3A). p53 was stabilized, and p21 and Puma expression were induced, in irradiated DN2 and HSC, indicating that DDR pathways were intact in these cell types *in vitro*. However, in contrast to HSC, p53 stabilization and induced expression of the pro-apoptotic protein, Puma, were transient in irradiated DN2 (Figure 3A). Furthermore, p21 expression was strongly induced in DN2 at early (1–4 h) time-points post IR whereas it was weakly induced in HSC at later time-points (12 and 24 h) (Figure 3A). Taken together, these results indicate that irradiated DN2 thymocytes rapidly induce the p53/p21 signaling cascade following DNA DSB generation which likely contributes to the ability of this cell type to induce a protective G_1_/S checkpoint, promoting their survival. Similar to previous observations seen in other radiosensitive cell types such as DP thymocytes (35), NH-HSCs appear to rapidly revert to inducing apoptosis, rather than protective DNA damage checkpoints, as evidenced by the long-term persistence of Puma and p53 stabilization in the presence of DNA DSBs (Figure 3A).

Western blot analysis of control DN2 and HSC whole cell extracts revealed that DN2 expressed higher endogenous levels of the DNA DSB sensor protein, ATM (Figure 3B) and of the DNA DSB repair *via* NHEJ proteins, DNA-PK_cs_ and 53BP1, than NH-HSCs (Figures 3B,C). The expression levels of the DNA DSB mediator protein, Chk2, were found to be comparable between DN2 and NH-HSCs, whereas endogenous levels of Rad51, a protein playing a key role in DNA DSB repair *via* homologous recombination was increased in NH-HSCs in comparison with DN2 cells (Figure 3C). In addition, compared with NH-HSC, DN2 cells were found to express higher levels of anti-apoptotic proteins, Bcl-2 and Bcl-_XL_, and of the pro-apoptotic protein, Bim (Figure 3D).

### Hypoxia Differentially Impacts on the DNA DSB Repair Capacity of DN2 Thymocytes and NH-HSCs

**Figure 4:**
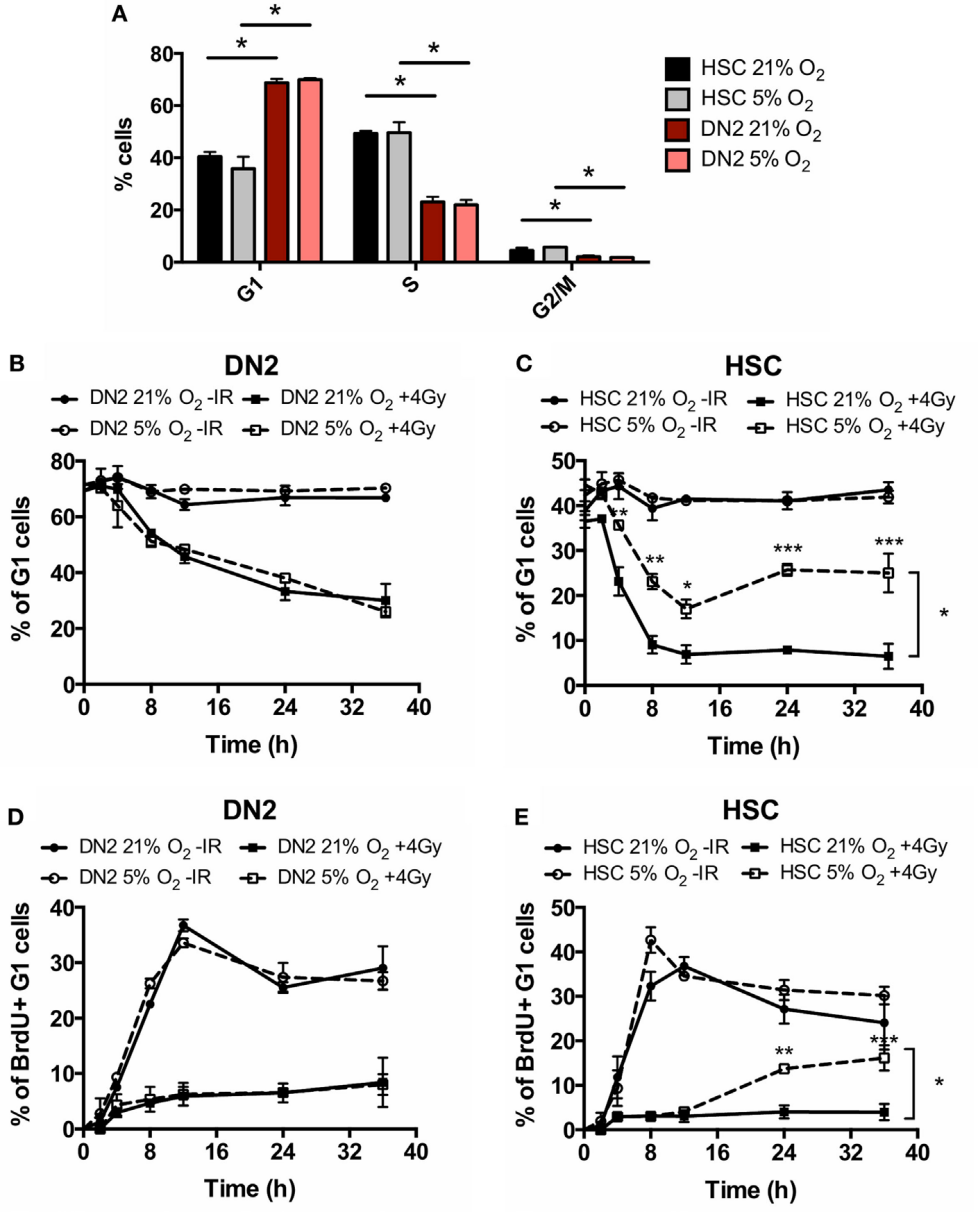
Cell cycle checkpoint activation of normoxic and hypoxic DN2 thymocytes. **(A)** Comparison of the percentage of cells in each phase of the cell cycle in NH-hematopoietic stem cell (HSC) and DN2 cells cultured in 21 or 5% O_2_. *p < 0.05, multiple *t*-tests with Holm–Sidak post-test correction. Quantification of average percentage of G_1_ phase **(B)** DN2 and **(C)** NH-HSC cells cultured in either 21 or 5% O_2_, 0–36 h post bromo-deoxyuridine (BrdU) pulse, with or without treatment with 4 Gy of ionizing radiation (IR). *p < 0.05, **p < 0.01, ***p < 0.001, two-way ANOVA analysis with Bonferroni post-test correction, *n* = 3. Quantification of average percentage of BrdU-labeled G_1_ phase **(D)** DN2 and **(E)** NH-HSC cells cultured in either 21 or 5% O_2_, 0–36 h post BrdU pulse, with or without treatment with 4 Gy of IR. **p < 0.01, ***p < 0.001, two-way ANOVA analysis with Bonferroni post-test correction, *n* = 3.

Our group has previously shown that the capacity of irradiated mouse MSCs and TECs to repair DNA DSBs is modulated by hypoxia, correlating with an effect on their intrinsic radioresistance (34, 40). Similar to the bone marrow, the thymus also consists of a hypoxic environment and therefore we were interested in investigating whether hypoxia may affect the radiobiology of DN2 thymocytes. To this end, we first investigated the effect of hypoxia on cell cycle checkpoint activation in irradiated DN2 thymocytes and NH-HSCs using the irradiation conditions studied previously. Culture in hypoxia did not affect the cell cycle progression of neither DN2 thymocytes nor NH-HSCs in normal growth conditions (Figure 4A). In addition, hypoxia exposure did not significantly impact on DNA damage checkpoint activation and recovery of DN2 thymocytes in response to irradiation (Figures 4B,D). However, culture under hypoxic conditions resulted in a significantly higher proportion of NH-HSCs being able to survive and resume the cell cycle, compared to their normoxic counterparts (Figures 4C,E; Figure S5 in Supplementary Material).

**Figure 5:**
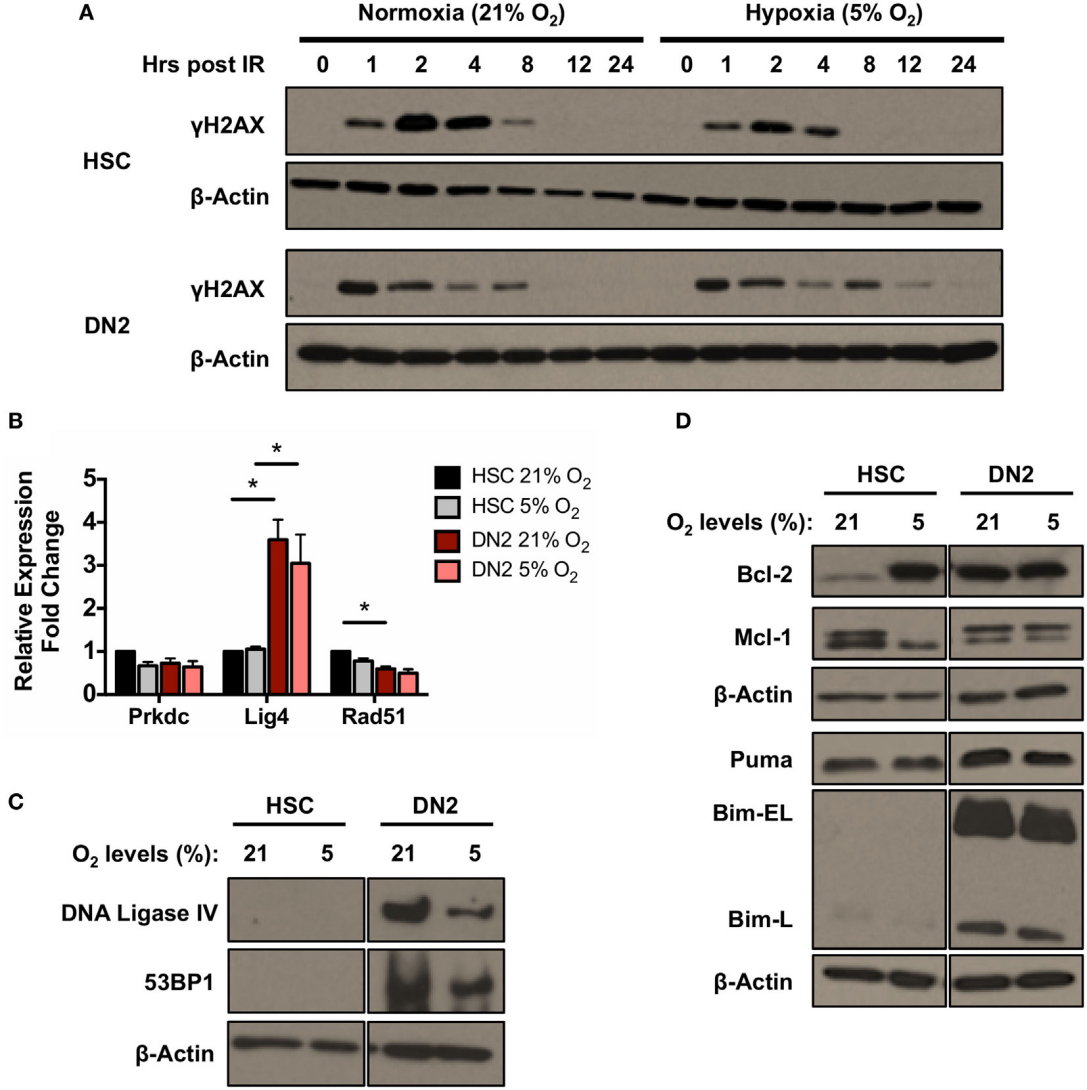
DNA repair and apoptotic proteins expression in normoxic and hypoxic NH-hematopoietic stem cell (HSC) and DN2 thymocytes. **(A)** Western blot analysis of H2AX (Ser139) phosphorylation in DN2 cells and NH-HSCs cultured in either normoxia (21% O_2_) or hypoxia (5% O_2_) at 0–24 h post 4 Gy irradiation. **(B)** mRNA expression levels of DNA repair factors DNA-PKcs (Prkdc), DNA Ligase IV (Lig4) and Rad51 in NH-HSC and DN2 cells in normoxia (21% O_2_) and hypoxia (5% O_2_). All values were normalized against β-actin and expressed relative to the NH-HSC normoxic sample. Representative western blots of **(C)** DNA damage response factors DNA ligase IV and 53BP1; and **(D)** pro- and anti-apoptotic proteins in NH-HSC and DN2 cells in normoxia (21% O_2_) and hypoxia (5% O_2_). All graphs show the average of three biological replicates. (*p < 0.05, multiple *t*-tests with Holm–Sidak post-test correction).

In addition, the kinetics of γH2AX induction and resolution in DN2 and NH-HSC cultured in normoxia and hypoxia was analyzed at different time-points post-irradiation (Figure 5A). The kinetics of γH2AX induction in both cell types at early time-points post-irradiation were found to be unaffected by oxygen tension (Figure 5A). However, in hypoxia, faster resolution of γH2AX phosphorylation was observed in the case of NH-HSCs, which may indicate faster repair of DNA DSBs. By contrast, although as previously shown the peak of γH2AX levels in DN2 cells occurs earlier, culture under hypoxic conditions results in slower kinetics of DSB repair in these cells, opposite to the results obtained with NH-HSCs.

In light of the previous results (Figure 3B), the effects of hypoxia on the endogenous expression levels of DNA repair and apoptotic factors in NH-HSCs and DN2 cells were also analyzed (Figures 5B,D). Culture under different oxygen levels did not cause significant changes in mRNA expression level of any of the DNA repair factors analyzed (DNA-PKcs, DNA ligase IV, and Rad51) in both cell types (Figure 5B). Similar to previous results (Figure 3B), DN2 thymocytes expressed (i) higher endogenous levels of DNA Ligase IV and 53BP1 and (ii) lower levels of Rad51 compared with NH-HSCs (Figures 5B,C). However, hypoxic DN2 cells had decreased levels of both proteins in comparison to their normoxic counterparts, correlating with the lower DSB repair efficiency detected in these cells in hypoxia (Figure 5A).

Protein levels of the anti-apoptotic factors Bcl-2 and Mcl-1 and the pro-apoptotic factors Bim and Puma were also analyzed. Interestingly, compared with normoxic cultures, culture of NH-HSCs under hypoxic conditions resulted in a large increase in endogenous Bcl-2 protein levels (Figure 5D). In line with this, the radioresistance of NH-HSCs was found to be increased in hypoxic conditions (Figure S5 in Supplementary Material). In normoxia, DN2 cells expressed high levels of endogenous Bcl-2 protein, similar to those of hypoxic NH-HSCs, but this was not affected by oxygen tension. Interestingly, NH-HSCs and DN2 cells showed different patterns of expression of the two Mcl-1 bands detected by Western blotting. While DN2 cells express higher levels of the upper band regardless of the oxygen levels, NH-HSC express equal levels of both bands in normoxia, but show preferential expression of the lower one in hypoxia (Figure 5D). With regard to the pro-apoptotic proteins, while the levels of Puma were similar between DN2 and HSC, DN2 showed much higher levels of the Bim-EL and Bim-L isoforms.

### *In Vivo* Response of DN Pro-T Cell Subpopulations to IR

**Figure 6:**
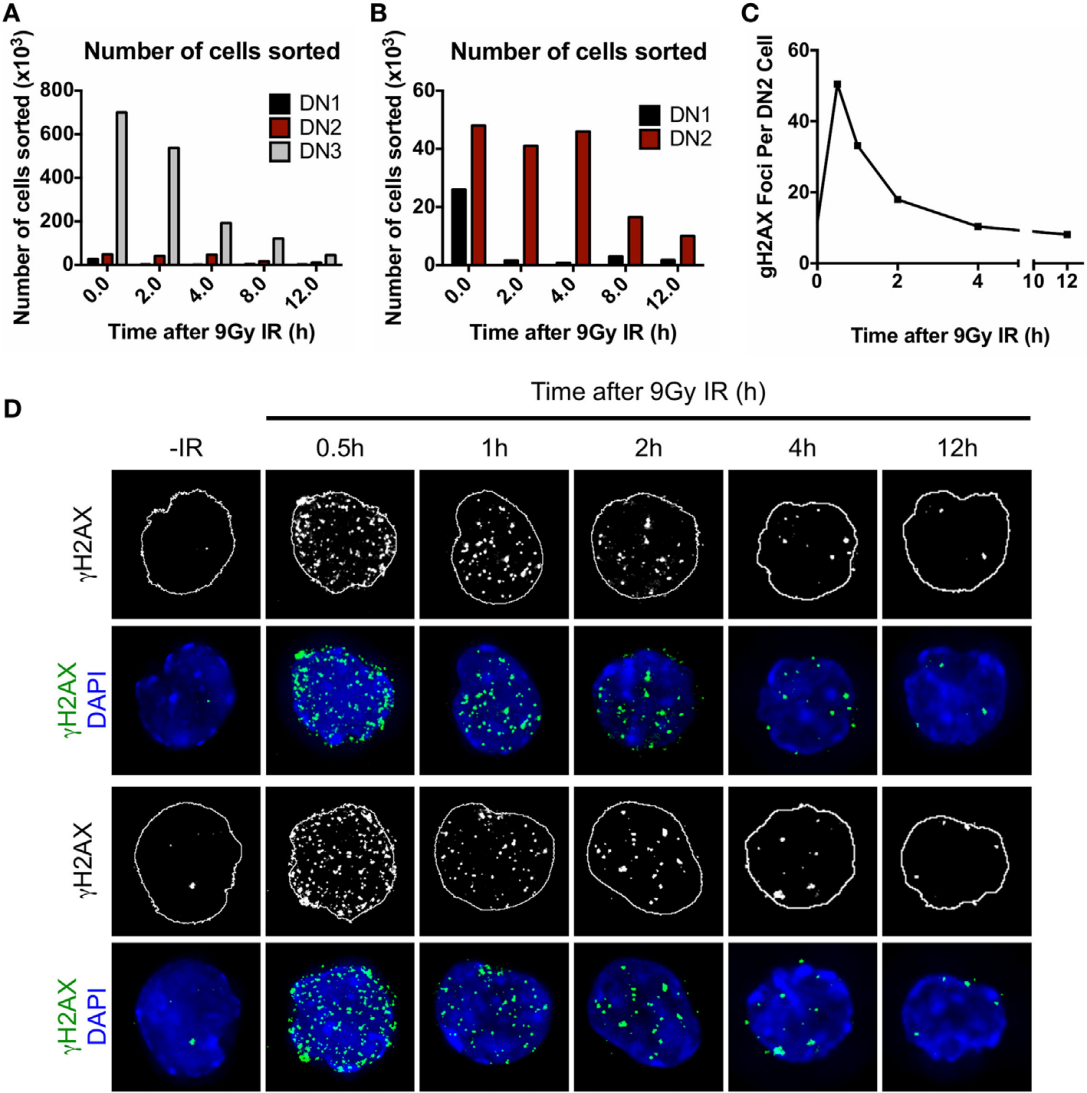
Characterization of DN pro-T cells *in vivo* response to ionizing radiation (IR). **(A)** Number of DN1, DN2, and DN3 pro-T cells; **(B)** number of DN1 and DN2 pro-T cells only that were recovered at different time points 0–12 h after irradiation with 9 Gy. **(C)** Average number of γH2AX IR-induced foci (IRIF) per nucleus and **(D)** representative images of DN2 nuclei stained for γH2AX IRIF and DAPI, corresponding to cells irradiated *in vivo* and isolated 0–12 h post IR.

Until now, we have demonstrated that the execution of the DDR and DNA DSB repair plays important roles in mediating the radioresistance of DN2 thymocytes *in vitro*. Therefore, our final objective was to determine whether DN2 thymocytes activate the DDR *in vivo* in response to irradiation. To do so, DN2 pro-T cells and their radiosensitive progenitors (DN1 cells) and progeny (DN3 cells) were isolated from either control or irradiated mice at different time-points following 9 Gy of whole body irradiation as graphically described in Figure S3A in Supplementary Material. The isolated cells were subsequently stained with specific antibodies to CD25, CD44, cKit, and CD3 and sorted into DN1, DN2, and DN3 subpopulations (Figure S3B in Supplementary Material). The numbers of both DN1 and DN3 pro-T cells recovered from the thymi dramatically dropped after IR treatment, whereas the number of DN2 pro-T cells remained higher in proportion at all time-points post-IR (Figures 6A,B). Interestingly, CD117 (cKit) surface expression by DN cells decreased over time following the IR treatment, an effect that was not correlated with changes in cell size, as measured by FCS measurements (Figure S6 in Supplementary Material). Sorted DN subpopulations were then analyzed at different time-points post IR for the appearance and resolution of γH2AX IRIF (Figures 6C,D). Similar to our findings *in vitro* (Figure 3A), *in vivo*-derived DN2 cells activated the DDR very quickly post IR, as evidenced by the peak in γH2AX IRIF formation at only 30 min post IR. Remarkably, irradiated DN2 cells rapidly resolved these DNA DSBs, as indicated by the resolution of the majority of γH2AX IRIFs by 4 h. As previously shown (Figures 6A,B), the numbers of DN1 and DN3 cells decreased dramatically post-IR due to cell death, and for DN1 cells, sufficient cells could not be recovered to carry out IRIF experiments. Since DN3 cells rapidly undergo apoptosis following IR, causing generalized γH2AX staining in their apoptotic nuclei, accurately quantifying γH2AX foci was not possible. In addition, it must be noted that all time-points referred to in this experiment correspond to the time when the thymi were isolated post IR and cell suspensions placed at 4°C. The time elapsed during sample preparation and sorting until cells were fixed (~2 h) is likely to underestimate the early kinetics of DNA damage repair in DN2 cells.

## Discussion

Following bone marrow transplantation (BMT), patients undergo a period of lymphopenia until their immune system is successfully regenerated. This lymphopenia renders them susceptible to life-threatening opportunistic infections and reactivation of endogenous viruses (41, 42). Although thymic cellularity drops drastically following irradiation due to the high radiosensitivity of the majority of thymocytes (15), many authors have reported a single wave of thymic auto-reconstitution occurring shortly after radiation exposure (16–20). Bosco et al. (21) determined that auto-reconstitution of the thymus was due to the survival of relatively radioresistant CD25^+^, CD44^+^, CD117^high^ conventional DN2 pro-thymocytes capable of recapitulating normal thymic differentiation and generating a cohort of cells exported to the periphery. In addition, they suggested that their mature peripheral T cell progeny may act as a first barrier against infections during the lymphopenic periods that follow BMT (21). In fact, it has been shown that in some cases, host-derived anti-cytomegalovirus-specific T cells are able to protect patients against viral infection during the lymphopenic period following BMT (43). As shown by Bosco et al. (21) in mouse BM chimeras, host-derived T cells survive for at least 6 months (21), display a polyclonal TCR Vβ repertoire, and appear to be functional both *in vitro* (17) and *in vivo* (21). To positively identify the DN2-derived cohort of T cells and distinguish them from their extra-thymically derived partners is difficult. However, since the thymic stroma is also irradiated in BM chimeras, it would be of interest to investigate the efficiency of negative selection of the TCR Vβ repertoire amongst host thymus-derived T cells.

The mechanisms that mediate the radioresistance of DN2 thymocytes, in contrast to radiosensitive DN1 and DN3 thymocytes, are so far poorly characterized. One of the main difficulties to overcome in studying the radiobiology of DN cells at the molecular level is their low number (especially DN1 and DN2) in the normal mouse thymus. Therefore, in this study, we utilized “*the Plastic Thymus*” culture system to expand purified DN2 pro-T cells *in vitro* to study the role of the DDR in mediating their radioresistance. Overall, this study has demonstrated that (i) DN2 thymocytes activate a rapid DDR in response to IR-induced DNA DSBs, which in turn, (ii) is likely to contribute to their ability to induce a protective G_1_/S checkpoint and thereby, provide DN2 cells with a time window for repairing DNA DSBs, promoting their survival.

Developing T cells undergo a process of rearrangement of their TCR *via* V(D)J recombination, which begins during the DN1 to DN2 transition (44). V(D)J recombination is initiated by Rag-mediated DNA DSBs (45) that trigger the activation of the DDR and the subsequent repair of the lesions *via* NHEJ (46–48). The DDR pathway is crucial for lymphocyte development as demonstrated by the fact that deficiency or loss-of-function mutations in important DDR genes such as ATM, Nbs1, 53BP1, TopBP1, and DNA ligase IV cause multiple defects in lymphocyte development and function, resulting in aberrant V(D)J rearrangements, lymphopenia, and increased susceptibility to hematological malignancies (49–54). Interestingly, in this study, compared with HSC, DN2 thymocytes were found to express higher endogenous levels of key DDR sensor and NHEJ proteins, including ATM, 53BP1, DNA ligase IV, and DNA-PK_cs_ (Figures 3B,C). This may reflect the need for DN2 thymocytes to have a robust DDR in place for effective regulation of TCR gene rearrangements during early thymic development. Also, in lymphocytes, ATM and p53 have been directly implicated in limiting DSBs caused by V(D)J recombination exclusively to the G_1_ phase of the cell cycle (55), *via* the repression of Cyclin D3 expression (56). This mechanism, which is specific for developing lymphocytes and not observed in mature T cells, may explain the preferential activation of the G_1_/S checkpoint in DN2 pro-T cells in response to DNA damage. However, the V(D)J recombination process not only occurs in DN2 cells but also in more advanced stages of T cell development, as well as in pro-B cells (10), which are highly radiosensitive. Therefore, gene rearrangement alone is not sufficient to explain the unique radioresistance of DN2 cells, compared with other lymphoid progenitors that also execute V(D)J recombination.

In addition, in comparison with HSCs, DN2 cells also expressed particularly high levels of the pro-apoptotic factor Bim (Figure 5D). Bim is an important apoptotic mediator in thymocyte biology that has been shown to be crucial in many stages of T cell development, such as for the regulation of lymphocyte progenitor survival and negative selection (57–60). Furthermore, Bim activity is known to be crucial for inducing apoptosis in thymocytes in response to IR (61), and it has been shown to be highly expressed in thymocytes prior to pre-TCR expression, that is, at the DN3 stage, and is downregulated upon signaling through the pre-TCR (62). Therefore, our observation of high levels of Bim being expressed in DN2 thymocytes is in line with the literature in the field. These high levels of Bim would be lethal for the cells if they were not counteracted by high expression of anti-apoptotic proteins such as Bcl-2 [also in line with our results (Figure 3B), which is also highly expressed in DN pro-T cells and is downregulated at the DP stage (63, 64)]. Interestingly, this high Bcl-2 expression is dependent on IL-7, an indispensable cytokine for T lymphopoiesis, whose receptor IL7Rα (CD127) is under the control of Notch1 signaling (65, 66), two of the main components in “*The Plastic Thymus*” culture system.

In contrast to DN2 pro-T cells, HSCs are highly radiosensitive (67, 68). Here, we have characterized the DDR of NH-HSCs cultured *in vitro* and compared it to that of DN2 cells, showing that NH-HSCs display higher radiosensitivity than DN2 cells particularly at high IR doses (closer to those used as cytoreductive regimens prior to BMT) (Figure 1). Several aspects of their biology may be contributing to this effect, such as (i) their slower activation of the DDR pathway in response to IR, (ii) lower levels of expression of DDR factors that are important for NHEJ, (iii) differential DNA damage checkpoint activation compared with DN2 cells (Figures 2 and 3), and (iv) more rapid cell cycle kinetics. It would appear that cell cycle kinetics alone cannot explain the increased susceptibility of NH-HSCs to irradiation. Strikingly, NH-HSCs from Bcl-2 transgenic mice, although retaining the same cell cycle kinetics as their non-Bcl-2 partners, did not undergo apoptosis following irradiation (unpublished observation). This indicates that the more rapid cell cycle kinetics of NH-HSC compared with DN2 cells was not responsible for their susceptibility to apoptosis. As previously shown by Bosco et al. (21), DN2 cells from Bcl-2 transgenic mice also showed dramatic changes in survival *in vitro*. Taken together, while the proliferation status of a given cell type may affect its radiosensitivity, we believe that our studies collectively suggest that the intrinsic orchestration of the DDR signaling pathway choice (i.e., induction of DNA damage checkpoints and DNA DSB repair versus cell death) plays a more important role in determining radiosensitivity. It would be of interest to compare the *in vitro* sensitivity of NH-HSC and DN2 thymocytes to glucocorticoids to help separate differences in their survival and DDR pathways. Previous experiments (20) had shown that host (presumably DN2)-derived thymocytes were seen in BM chimeras where recipient mice had been treated with hydrocortisone acetate 48 h prior to irradiation and BM reconstitution. This suggests that DN2 cells are glucocorticoid resistant *in vivo*. Animals treated with glucocorticoids and left to recover showed no sign of hematopoietic failure, suggesting indirectly that HSC and/or multipotent progenitors do not appear to be glucocorticoid sensitive *in vivo* (20).

Interestingly, culturing NH-HSCs under hypoxic conditions (5% O_2_) increased the efficiency of DNA repair (Figure 5A) and suvival capacity (Figure 4; Figure S5 in Supplementary Material) in these cells, as well as inducing an important up-regulation of Bcl-2 protein levels (Figure 5D). By contrast, culture of DN2 cells in hypoxia resulted in less efficient DNA repair (Figure 5A) and decreased levels of DNA ligase IV and 53BP1 (Figure 5C) were detected in this condition. However, surface marker analysis of normoxic and hypoxic *in vitro* DN2 cultures demonstrated that, while DN2 cells cultured in normoxia maintain a stable phenotype, culturing the same cells in hypoxia results in the accumulation of a CD25^−^ subpopulation that increases in number over time (data not shown). This fact makes it impossible to compare the DDR of DN2 cells in normoxia and in hypoxia, since in this last condition, a mixed population of CD25^+^ DN2-like cells and CD25^−^ cells of unknown nature are analyzed jointly and it is impossible to distinguish the specific contributions of each one to the overall result.

Despite these previous observations, DN2 pro-T cells cultured *in vitro* under normoxia retain many of their phenotypic and functional characteristics, which makes this culture system a very useful tool for the study of DN2 cells at the molecular level. For this reason, it was important to also investigate whether the robust DDR activation and DNA DSB repair by DN2 thymocytes observed *in vitro* also occurs within the thymus itself (Figure 6). Cell numbers recovered from the thymus at different time-points post IR indicated that a higher proportion of DN2 thymocytes remain viable, in comparison with DN1 and DN3 thymocytes, whose numbers dropped quickly following IR exposure (Figures 6A,B). Surprisingly, cKit surface expression by DN cells was observed to decrease over time after IR (Figure S6 in Supplementary Material). Previous studies (69) have indicated that *ckit* is a target gene for Notch signaling. Therefore, the decrease in CD117 expression may be the result of decreased Notch ligand expression by TECs following irradiation (34).

Interestingly, DN2 pro-T cells displayed discrete, single, γH2AX IRIF in the absence of IR treatment (Figure 6D), which according to the observations from Chen et al. (52), may correspond to sites of V(D)J recombination-induced DSBs. Similar to the results obtained from DN2 cells *in vitro*, DN2 thymocytes *ex vivo* displayed fast activation of their DDR following IR, with γH2AX IRIF numbers peaking at 30 min post-IR. The quick disappearance of γH2AX IRIF, which was largely complete by 4 h after IR, is an indicator of the high efficiency in DSB repair displayed by DN2 cells *in vivo*. This is, to the best of our knowledge, the first time that the *in vivo* DNA repair efficiency of DN2 cells has been characterized.

In conclusion, we have used “*The Plastic Thymus*” *in vitro* culture system to investigate in detail the mechanisms responsible for the relatively high DN2 radioresistance that allows thymic auto-reconstitution following radiation exposure. We have shown that multiple facets of their DDR are likely to contribute to their enhanced survival in contrast to radiosensitive HSCs. Finally, we have demonstrated that DN2 pro-thymocytes are capable of efficiently repairing DNA DSBs *in vivo* following lethal thymic irradiation.

## Ethics Statement

All animal experiments were carried out within institutional guidelines (authorization numbers 1886 and 1888 from Kantonales Veterinäramt, Basel).

## Author Contributions

IC-A and TS: conception and design, collection and assembly of data, data analysis and interpretation, and manuscript writing. NB: conception and design, data analysis and interpretation, manuscript writing, and final approval of manuscript. AR: conception and design, financial support, data analysis, and interpretation. RC: conception and design, financial support, data analysis and interpretation, manuscript writing, and final approval of manuscript.

## Conflict of Interest Statement

Co-author NB is currently employed by Nestlé Research Center Asia. All other authors declare no competing interests. Co-author TS declares her affiliation with Frontiers and the handling Editor states that the process nevertheless met the standards of a fair and objective review. All other authors declare they have no potential conflicts of interests to disclose.
