# Supplementary material for: DN2 Thymocytes Activate a Specific Robust DNA Damage Response to Ionizing Radiation-Induced DNA Double-Strand Breaks
Source: Front Immunol. 2018 Jun 11;9:1312. doi: 10.3389/fimmu.2018.01312 (PMC6004388; doi:10.3389/fimmu.2018.01312)
Supplement: Supplementary file 1 [file data_sheet_1.PDF]

Supplementary Material

DN2 thymocytes activate a specific robust DNA Damage response to IR-induced DNA double-strand breaks

Irene Calvo-Asensio[1], Tara Sugrue[1], Nabil Bosco[2], Ton Rolink[3] & **Rhodri Ceredig[1*]**

[1]*National University of Ireland, Galway,* [2]*Nestle R&D Center (Pte) Ltd, 29 Quality Rd, Singapore 618802* [3]*Department of Biomedicine, University of Basel.*

Corresponding Author: Rhodri Ceredig
rhodri.ceredig@nuigalway.ie

1 Supplementary Methods

The 'Plastic Thymus' Culture System

The *'Plastic Thymus'* culture system was set up as previously described (1). In short, wells of a 6-well culture plate were coated overnight at 4°C with 2 ml of a 10 µg/ml solution of mouse monoclonal anti-human IgG_1 Fc antibody (HUF5.4) diluted in sterile PBS (Sigma-Aldrich) (Supplementary Figure 1). The PBS containing HUF5.4 was then removed and wells washed twice with 5 ml of IMDM growth medium prior to the addition of DL4-Fc diluted in growth medium at a final concentration of 2 µg/ml. 2 ml of this DL4-Fc solution were added per well and incubated overnight at 4°C. The wells were then washed twice with growth medium in order to eliminate any free DL4-Fc molecules not bound to the well surfaces. Sorted DN2 thymocytes were cultured in 2 ml Serum Free-Iscove's Modified Dulbecco's Medium (SF-IMDM) (Gibco) supplemented with 5% HyClone FCS (Thermo Scientific), 10% IL-7-containing supernatant, 0.1 µg/ml SCF and 0.2% Ciproxin® (Bayer Pharmaceutical) per well at 37°C in 21% or 5% O_2.

Isolation and sorting of mouse CD4/CD8 double negative (DN) 1-3 sub-populations

For the DN cell isolation, thymi were crushed through a 70µm pore size cell strainer in sterile IMEM (Gibco) to generate a thymocyte suspension. CD4+ and CD8+ T lymphocytes depletion was achieved

by incubating the thymocyte suspension with culture supernatants of rat IgM anti-mouse-CD4 (RL172.4) and rat IgM anti-mouse-CD8 monoclonal antibodies (3.168.8.1) (2,3) and lysing them using Low-Tox®-M Rabbit Complement (Cedarlane®). Viable DN thymocytes were then separated from the cell debris using Ficoll-Hypaque density media (Sigma-Aldrich) and stained with anti-CD25, anti-CD44 and anti-CD117 antibodies at 4°C for 30 minutes in IMDM culture medium. DN thymocytes were then re-suspended in 500 µl of IMDM following a washing step and filtered prior to sorting using a BD FACS Aria II® (BD Biosciences). Dead cells were excluded using propidium iodide staining.

Antibodies

For western blotting, anti-phospho-Histone H2A.X (Ser139) mouse monoclonal antibody (Millipore), anti-H2AX rabbit polyclonal antibody, anti-ATM [2C1(1A1)] mouse monoclonal antibody, anti-DNA-PKcs (Y393) rabbit monoclonal antibody, anti-DNA Ligase IV rabbit polyclonal antibody, anti-Rad51 rabbit polyclonal antibody (Abcam), anti-ATR (N-19) goat polyclonal antibody, anti-Chk2 (H-300) rabbit polyclonal antibody, anti-p53 (1C12) mouse monoclonal antibody, anti-Bcl-XL (54H6) rabbit monoclonal antibody, anti-Bcl-2 (D17C4) rabbit monoclonal antibody, anti-Bim (C34C5) rabbit monoclonal antibody, anti-Puma (E1S7A) rabbit polyclonal antibody, anti Mcl-1 (D35A5) Rabbit monoclonal antibody, anti-β-Actin rabbit polyclonal antibody (Sigma-Aldrich), Pierce horseradish peroxidase (HRP)-conjugated rabbit anti-mouse IgG antibody, and Immuno-Pure HRP-conjugated goat anti-rabbit IgG antibody (Thermo Scientific), were used. For immunofluorescence staining, anti-phospho-Histone H2A.X (Ser139) mouse monoclonal antibody (Millipore) and fluorescein (FITC)-conjugated AffiniPure F(ab0)2 Fragment goat anti-mouse IgG antibody (Jackson ImmunoResearch Laboratories Inc.) were used. For flow cytometry, anti-5-bromodeoxyuridine (BrdU) mouse monoclonal antibody (BD Biosciences), FITC-conjugated anti-mouse IgG (whole molecule) antibody (Sigma-Aldrich), PE-conjugated anti-CD3ε (145-2C11) (Biolegend), PE- or APC-conjugated anti-CD117 (cKit) (2B8) (BD Biosciences), and PE-Cy7- or APC-conjugated anti-CD44 (IM7) (BD Bioscience) were used.

2 Supplementary Figure Legends

Supplementary Figure 1: ***DN2 and HSC in vitro culture systems.*** **(A)** "The plastic thymus" culture system: Tissue culture wells used for DN2 thymocyte culture were coated with DL4-Fc fusion protein bound to the well surface using with anti-human IgG1-Fc antibody (HUF5.4) In addition, culture medium was supplemented with Interleukin-7 (IL-7) and Stem Cell Factor (SCF) (1). **(B)** NH-HSC generation and culture system: retroviral transduction of the NUP98-HOXB4 fusion construct allows long-term expansion of HSCs in vitro. Culture medium is supplemented with Interleukin-6 (IL-6) and Stem Cell Factor (SCF) (4).

Supplementary Figure 2: ***DN2 phenotype remains stable over time in culture.*** Representative cytograms of DN2 cells stained for their surface expression of **(A)** CD25 and CD117 (cKit); and **(B)** CD25 and CD44, after 5 and 12 days of normoxic (21% O2) culture.

Supplementary Figure 3: ***Isolation and analysis of DN pro-T cells following IR treatment.*** **(A)** DN2 pro-T cell isolation and enrichment protocol [adapted from (5)] and **(B)** sorting strategy used for the purification of mouse DN pro-T cells from control and irradiated mice.

Supplementary Figure 4: ***Cell cycle progression of DN2 pro-T cells and NH-HSC cells.*** Representative cytograms of control un-irradiated DN2 and NH-HSC cells, 0-24h after BrdU labelling and stained for BrdU incorporation and DNA content (propidium iodide). Representative gating strategy for the identification of G_1, S and G_2/M phase cells is shown.

Supplementary Figure 5: ***NH-HSCs display a higher radio-resistance in hypoxia.*** Clonogenic survival assays of NH-HSCs γ-irradiated at 0.5-4 Gy and cultured for 3 days in normoxia (21% O_2) or hypoxia (5% O_2). Error bars represent mean ± SD, n=4. * $p<0.05$, Two-way ANOVA analysis.

Supplementary Figure 6: ***DN1/2 pro-T cells downregulate surface levels of cKit upon irradiation.*** **(A)** Representative cKit staining and **(B)** MFI of DN1/2 thymocytes 0 to 12h after irradiation with 9Gy.

3 Supplementary References

1. Gehre N, Nusser A, von Muenchow L, Tussiwand R, Engdahl C, Capoferri G, Bosco N, Ceredig R, Rolink AG. A stromal cell free culture system generates mouse pro-T cells that can reconstitute T-cell compartments in vivo. *Eur J Immunol* (2015) **45**:932–942. doi:10.1002/eji.201444681
2. Ceredig R, Sekaly RP, MacDonald HR. Differentiation in vitro of Lyt 2+ thymocytes from embryonic Lyt 2- precursors. *Nature* (1983) **303**:248–50.
3. Ceredig R, Lowenthal JW, Nabholz M, MacDonald HR. Expression of interleukin-2 receptors as a differentiation marker on intrathymic stem cells. *Nature* (1985) **314**:98–100.
4. von Muenchow L, Engdahl C, Karjalainen K, Rolink AG. The selection of mature B cells is critically dependent on the expression level of the co-receptor CD19. *Immunol Lett* (2014) **160**:113–119. doi:10.1016/j.imlet.2014.01.011
5. Ceredig R, Rolink T. A positive look at double-negative thymocytes. *Nat Rev Immunol* (2002) **2**:888–897. doi:10.1038/nri937
