# Supplementary figures and images for: DN2 Thymocytes Activate a Specific Robust DNA Damage Response to Ionizing Radiation-Induced DNA Double-Strand Breaks
Source: Front Immunol. 2018 Jun 11;9:1312. doi: 10.3389/fimmu.2018.01312 (PMC6004388; doi:10.3389/fimmu.2018.01312)

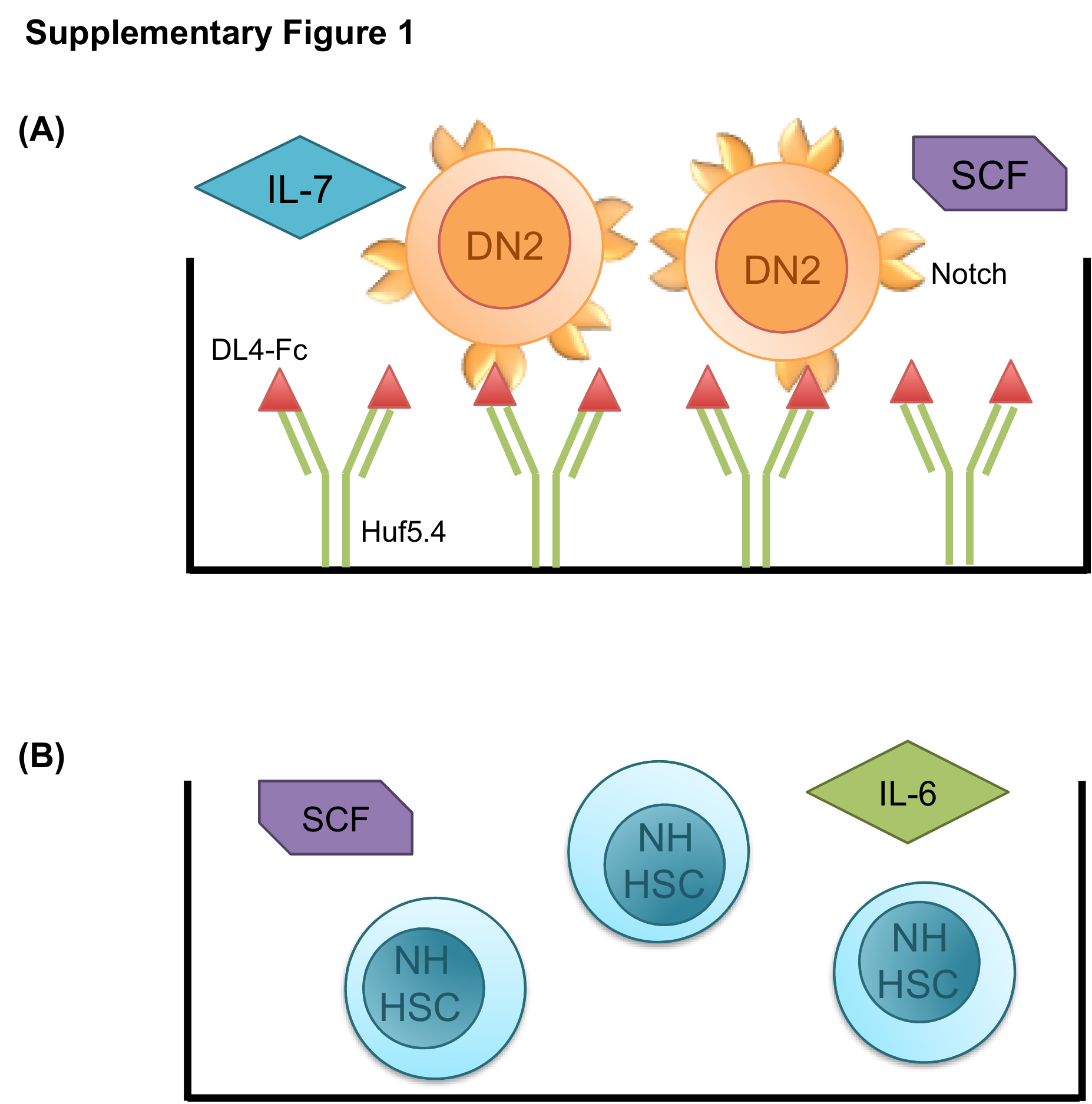

Supplement: Supplementary file 2 [file image_1.tiff]

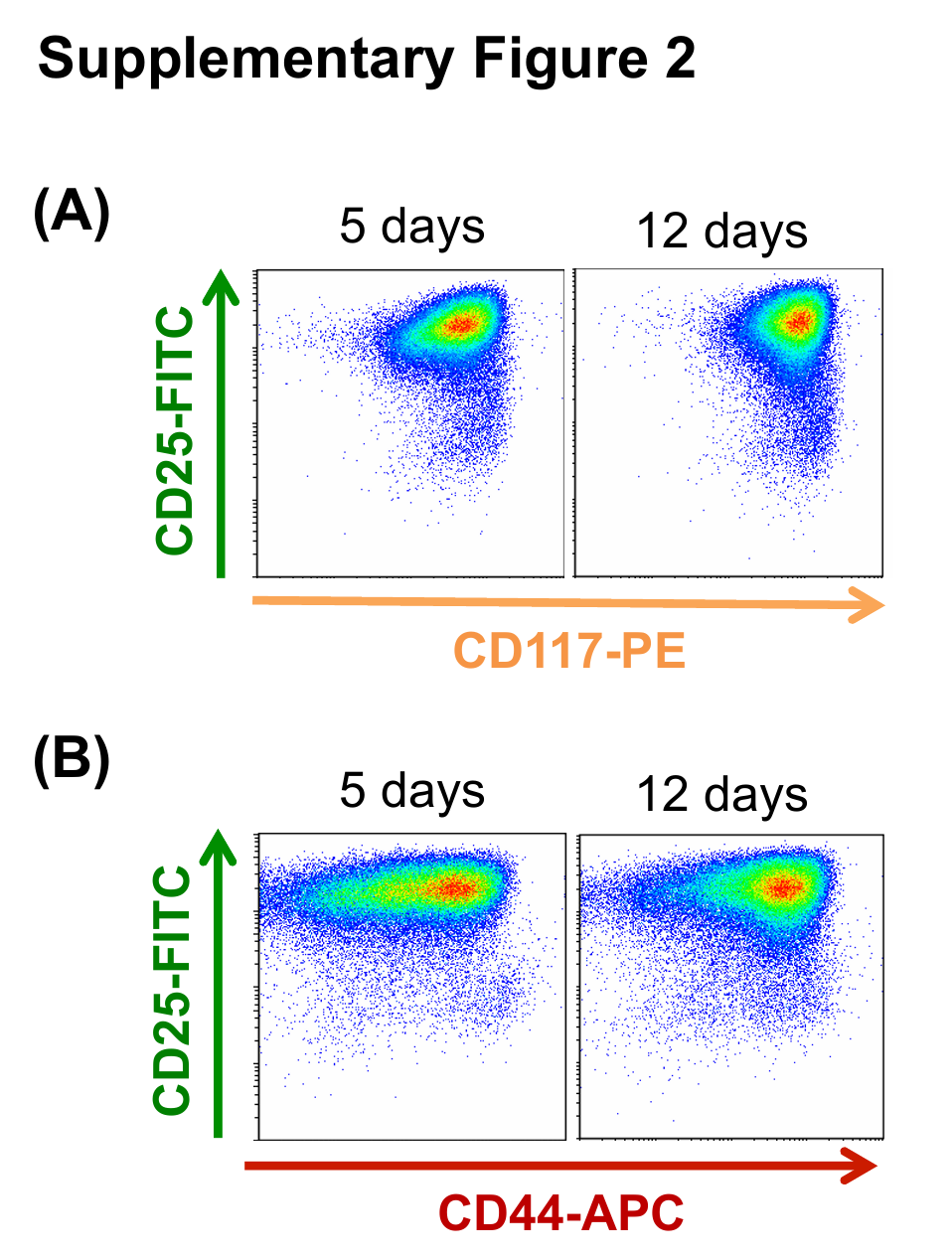

Supplement: Supplementary file 3 [file image_2.tiff]

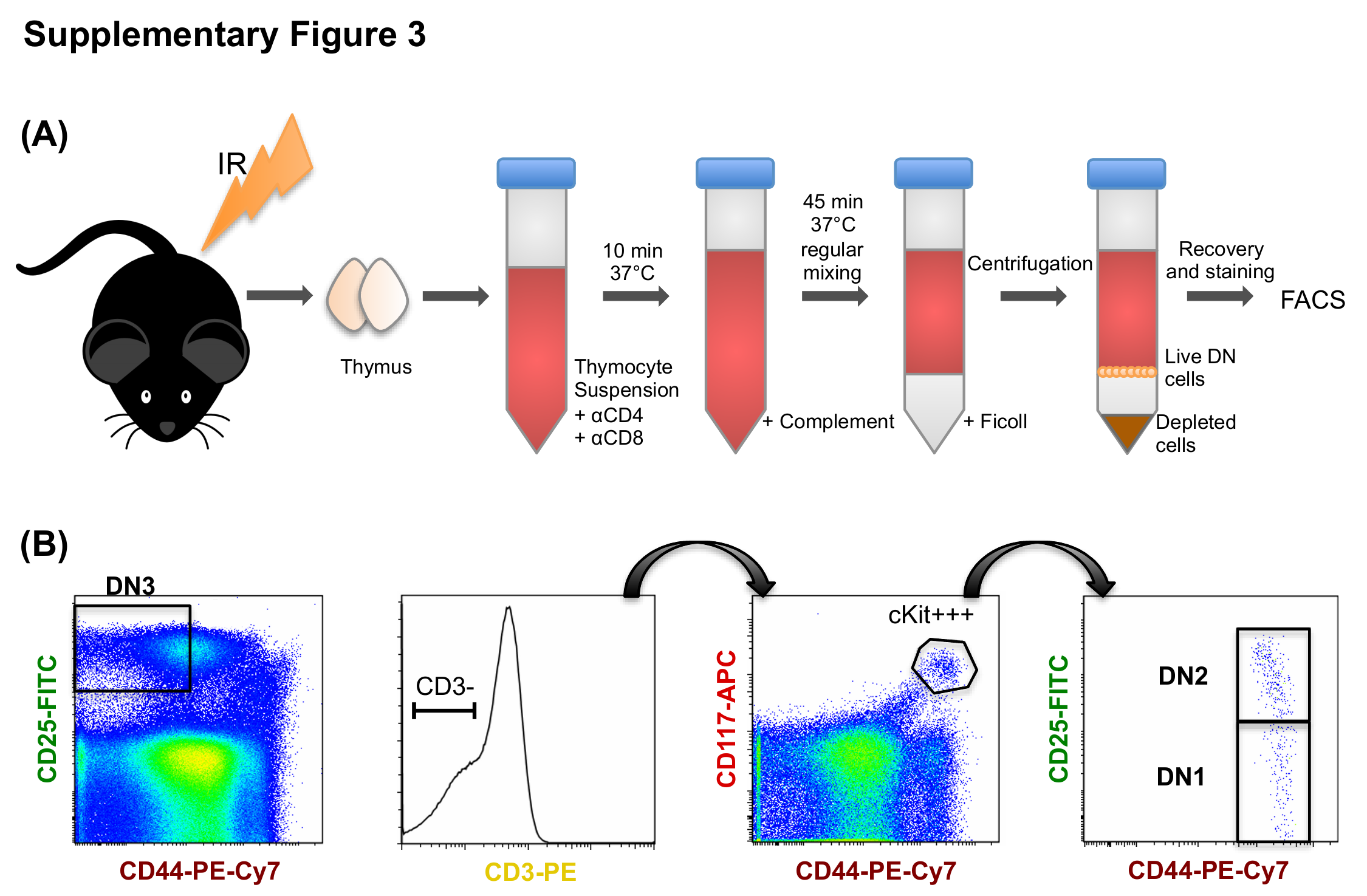

Supplement: Supplementary file 4 [file image_3.tiff]

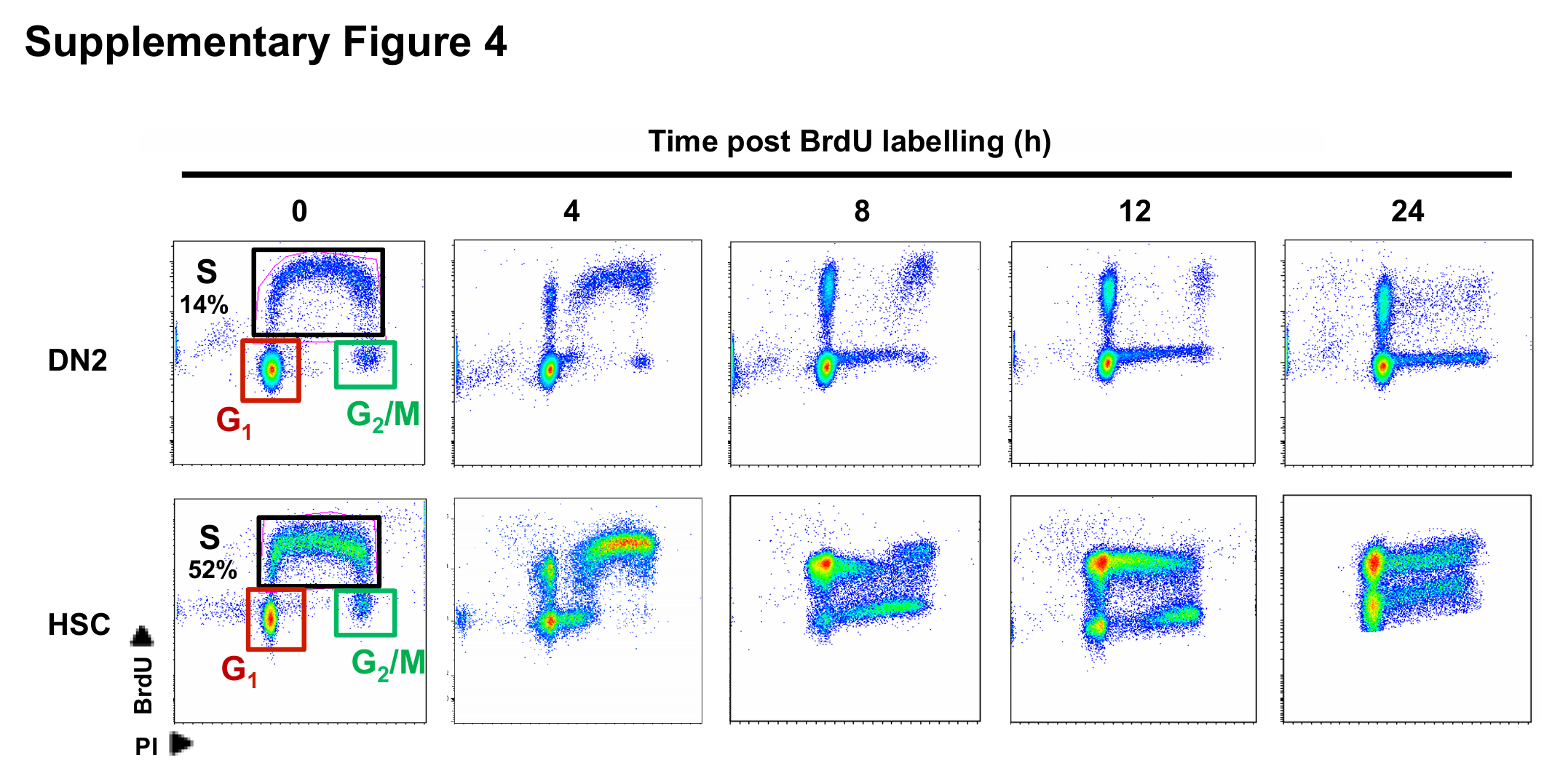

Supplement: Supplementary file 5 [file image_4.tiff]

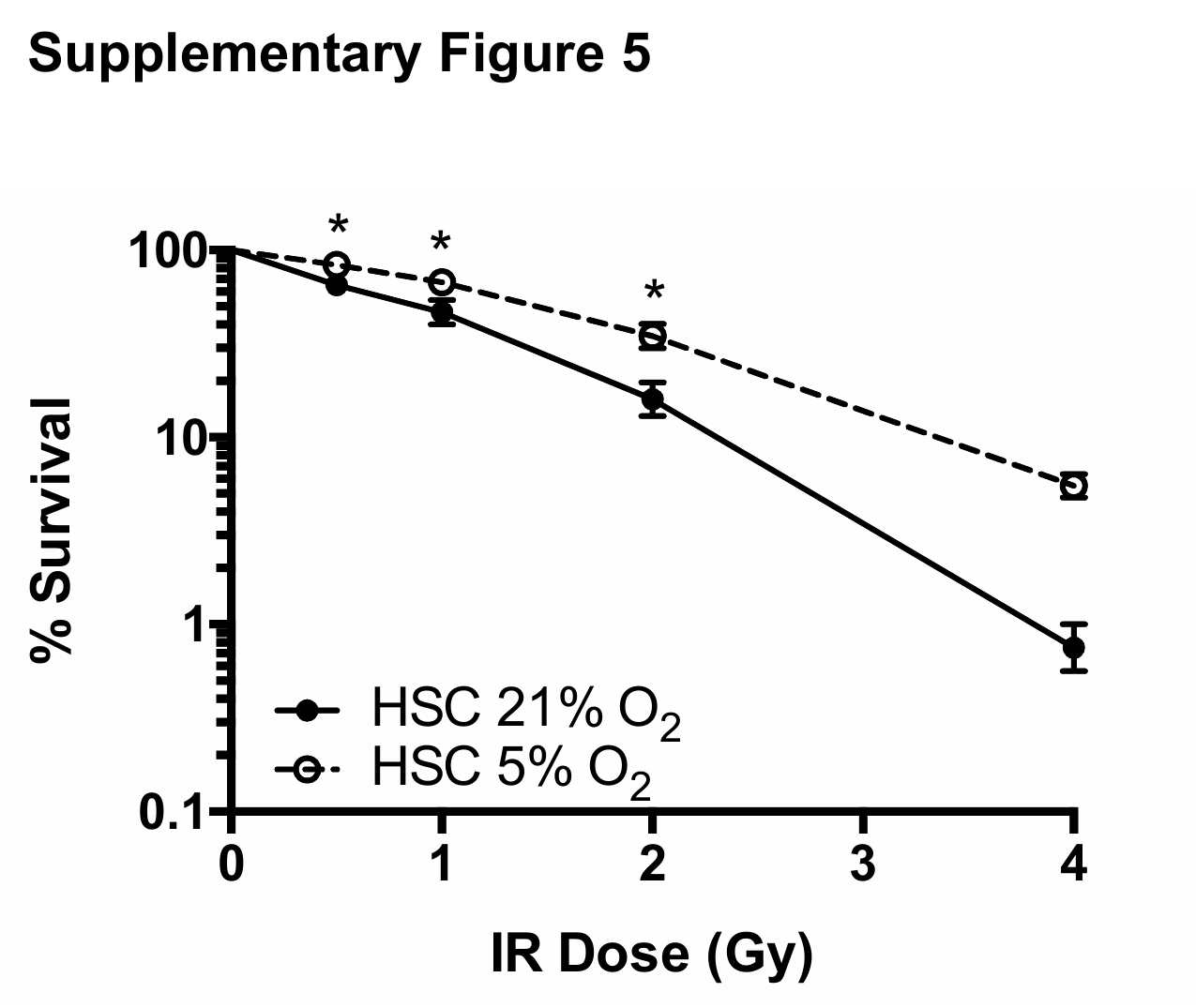

Supplement: Supplementary file 6 [file image_5.tiff]

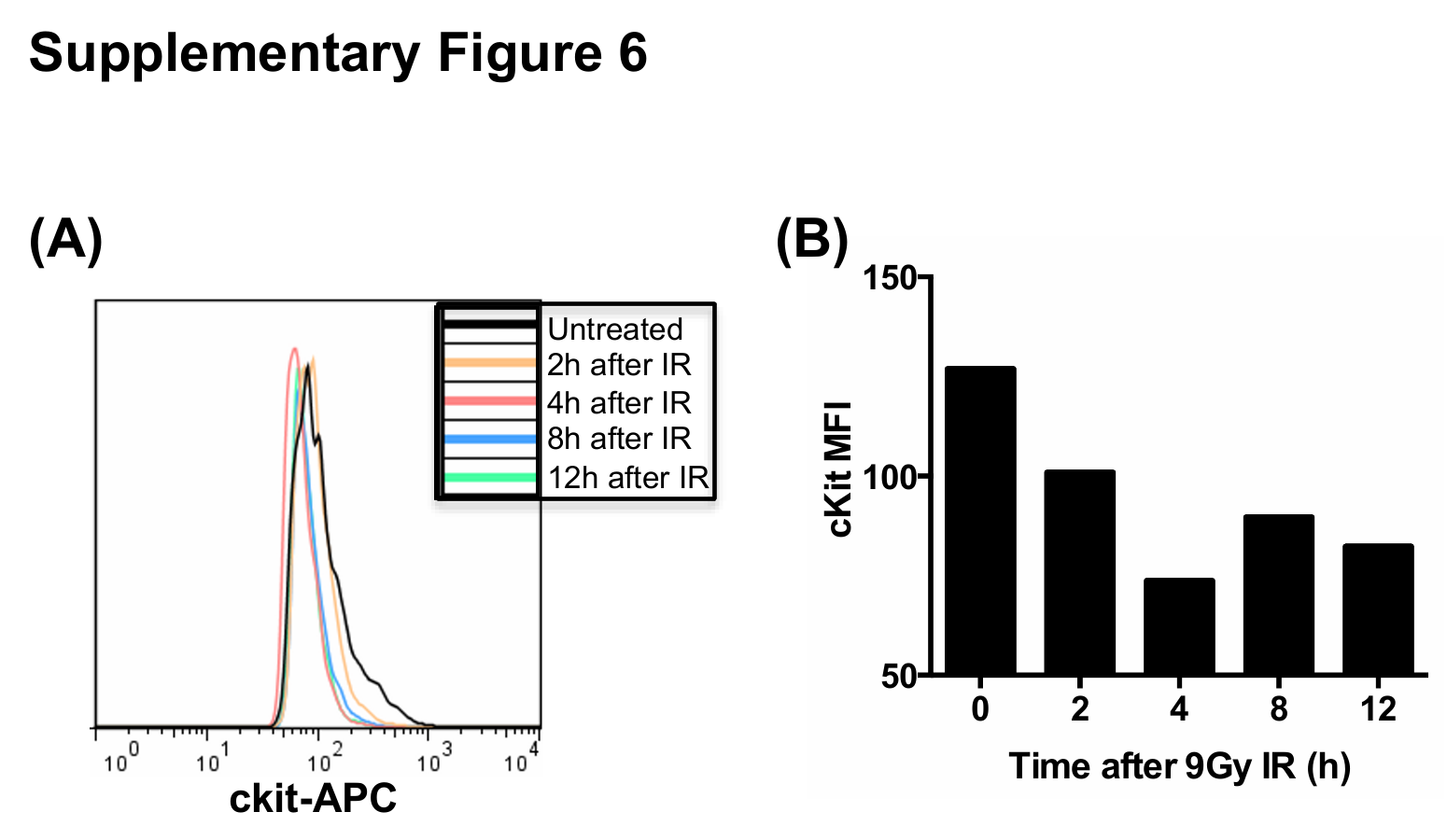

Supplement: Supplementary file 7 [file image_6.tiff]
